# Implications of accounting for management intensity on carbon and nitrogen balances of European grasslands

**DOI:** 10.1371/journal.pone.0201058

**Published:** 2018-08-13

**Authors:** Jan Blanke, Niklas Boke-Olén, Stefan Olin, Jinfeng Chang, Ullrika Sahlin, Mats Lindeskog, Veiko Lehsten

**Affiliations:** 1 Lund University, Department of Physical Geography and Ecosystem Science, Sölvegatan 12, 223 62 Lund, Sweden; 2 Laboratoire des Sciences du Climat et de l’Environnement, UMR8212, CEA-CNRS-UVSQ, Gif-sur-Yvette, France; 3 Lund University, Center for Environmental and Climate Research, Sölvegatan 37, 223 62 Lund, Sweden; 4 Swiss Federal Institute for Forest, Snow and Landscape research (WSL), Zürcherstr. 11, CH-8903 Birmensdorf, Switzerland; Fred Hutchinson Cancer Research Center, UNITED STATES

## Abstract

European managed grasslands are amongst the most productive in the world. Besides temperature and the amount and timing of precipitation, grass production is also highly controlled by applications of nitrogen fertilizers and land management to sustain a high productivity. Since management characteristics of pastures vary greatly across Europe, land-use intensity and their projections are critical input variables in earth system modeling when examining and predicting the effects of increasingly intensified agricultural and livestock systems on the environment. In this study, we aim to improve the representation of pastures in the dynamic global vegetation model LPJ-GUESS. This is done by incorporating daily carbon allocation for grasses as a foundation to further implement daily land management routines and land-use intensity data into the model to discriminate between intensively and extensively used regions. We further compare our new simulations with leaf area index observations, reported regional grassland productivity, and simulations conducted with the vegetation model ORCHIDEE-GM. Additionally, we analyze the implications of including pasture fertilization and daily management compared to the standard version of LPJ-GUESS. Our results demonstrate that grassland productivity cannot be adequately captured without including land-use intensity data in form of nitrogen applications. Using this type of information improved spatial patterns of grassland productivity significantly compared to standard LPJ-GUESS. In general, simulations for net primary productivity, net ecosystem carbon balance and nitrogen leaching were considerably increased in the extended version. Finally, the adapted version of LPJ-GUESS, driven with projections of climate and land-use intensity, simulated an increase in potential grassland productivity until 2050 for several agro-climatic regions, most notably for the Mediterranean North, the Mediterranean South, the Atlantic Central and the Atlantic South.

## 1 Introduction

Managed grassland is one of the dominant forms of land-use in Europe, covering 80 million hectares or 22% of the EU-25 land area (EEA, 2005). At the same time, European managed grasslands are amongst the most productive in the world. It is estimated that grassland biomass harvested for forage production, which is a fraction of net primary productivity (NPP), is in the range of 2-8 t C ha^−1^ yr^−1^ [1] and approaches NPP of European temperate forests [2]. While the productivity of natural grasslands is regulated mostly by temperature and the amount and timing of precipitation [3], grass production in Europe is to a large extent also controlled by applications of nitrogen (N) fertilizers and land management in general to sustain a high productivity. The evaluation of regional and country-specific fertilizer data has shown that the comparatively high productivity of European grasslands is also accompanied by high applications of mineral fertilizer [4]. The dominant role of management practices has also been demonstrated by [5] who analyzed 38 manipulative grassland experiments and found that combinations of land management practices remained the dominant set of factors in determining the growth of grassland plant communities.

Similar to cropland and forest management intensity [6, 7], grassland management intensity is not uniform across Europe since agricultural grasslands include silage and hay fields, pastures under intensive production, as well as semi-natural grasslands [8]. As with arable systems, ecological problems are mostly associated with the most productive grassland types. Impacts resulting from intensively managed grassland include nutrient leakages from inadequate nutrient management, soil erosion due to high stocking densities, and particularly poor biodiversity. Since the character of agricultural areas and land-use vary greatly across the world, land-use intensity becomes a critical variable and characteristic of managed land systems for use in earth system modeling when examining and predicting the negative effects of increasingly intensified agricultural and livestock systems on the environment [9, 10]. Since land management may be critical for projected responses to future climate change and elevated CO_2_ in models of ecosystem function and primary production, projections of land-use intensity need to be considered [5, 11]. For grasslands for example, recent changes to the Common Agricultural Policy (CAP) are likely to lead to a continuing trend towards intensification of dairy farming. The area of grasslands instead is projected to decrease [12].

One of the tools to estimate the ecosystem functions of grasslands in the future are dynamic global vegetation models (DGVMs). They are used for exploring and predicting the coupled dynamics of ecosystem functioning, climate-carbon cycle interactions and biome distributions [13]. Recent development strands in some DGVMs are now addressing more advanced large-area representations of land-use change, land management functionalities and N cycling [14, 15]. However, while different land cover types such as croplands, pastures and forests and their historical and scenario-based fractions are represented now in many DGVMs and earth system models, land-use intensity is still neglected in many models or for certain land cover types [10]. This is also true for the DGVM LPJ-GUESS [15, 16].

While in LPJ-GUESS, cropland management intensity has been incorporated recently [17], this is not the case for pastures, which are merely driven by climate, CO_2_ and nitrogen deposition. In this study, we aim to improve the representation of pastures in LPJ-GUESS by incorporating a recently developed daily allocation for grasses as a foundation to further implement daily land management routines as well as land-use intensity data to discriminate between intensively and extensively used regions. We further compare simulations of this updated version (from this point called LPJ-GUESS-LUI) with leaf area index (LAI) observations, reported regional grassland productivity and simulations conducted with ORCHIDEE-GM [18]. Additionally, we analyze the implications of including pasture fertilization and daily management for the present and for future projections.

## 2 Material & methods

### 2.1 LPJ-GUESS

LPJ-GUESS is a well-established, process-based ecosystem model designed for regional to global applications. Vegetation is modeled via plant functional types (PFTs) which represent the globally most abundant growth strategies. These can be distinguished in terms of e.g. growth form, phenology, life history strategy, allometry, photosynthetic pathway and a limited set of bioclimatic limits. Soil C and N dynamics are based on the CENTURY model [19] which contains eleven soil organic matter (SOM) and litter pools differing in their C to N (C:N) ratios and decay rates. A detailed description of the model is given by [16] while the N cycle implementation including the N allocation algorithm is described in [15]. Recent model developments include the incorporation of land-use change dynamics together with a crop module [14] which is based on approaches by [20] and [21]. [17] updated this version of the crop module by incorporating nitrogen dynamics and C-N interactions also for crops. Managed grasslands are represented in the standard version of LPJ-GUESS by removing 50% of the above-ground carbon [14]. This is meant to represent a 90% removal in intensively grazed pastures and a 50% re-entering of this carbon back to the litter pool as manure. The carbon allocation for grasses is done at the end of the year in the standard version of LPJ-GUESS.

#### 2.1.1 Daily carbon allocation for grass PFTs

**Fig 1:**
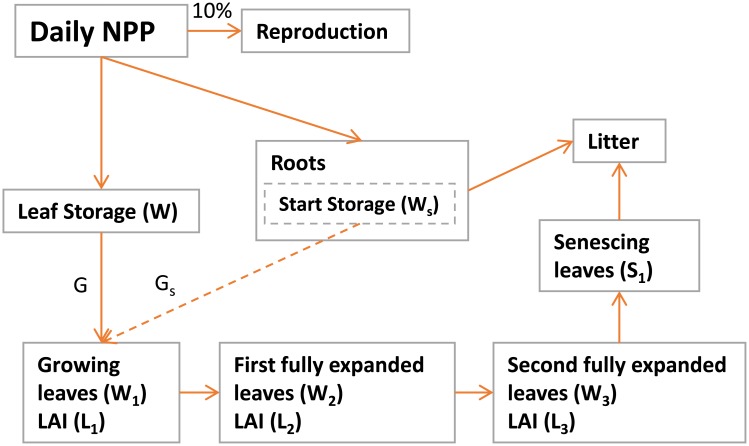
Flowchart describing the carbon pools used for the daily carbon allocation model for grasses on a daily time scale. *G* denotes the carbon flux from leaf storage while *G*_*s*_ denotes the carbon flux from the start storage.

To implement daily pasture management regimes and allow realistic feedbacks between management and vegetation, we incorporated the daily carbon allocation routine for natural C3 and C4 grasses developed by [22] into LPJ-GUESS (described above). The implemented functions and model modifications are based on theory from [23]. Carbon assimilated by photosynthesis on a daily time step is allocated dynamically to one root and four different shoot biomass compartments (growing leaves, first fully expanded leaves, second fully expanded leaves, senescing leaves), or is respired by autotrophic processes (see Fig 1). At the end of each simulation day, 10% of NPP is transferred into the reproduction pool while the rest is separated into leaf storage, roots and start storage. Thereby, the start storage enables growth when the grass has a low LAI but the conditions are favorable. Carbon moves from leaf storage to the four biomass compartments differing in leaf age classes based on a temperature dependent growth factor and daily phenology. The daily phenology is calculated as the minimum value of the ratio between water supply and water demand for full leaf cover, and the ratio between growing degree days above 5 degree Celsius (gdd5) and the lifeform specific gdd5 sum for full leaf cover. The movement between the four shoot biomass compartments is controlled by a PFT specific transfer rate and temperature.

#### 2.1.2 Pasture management

After incorporating daily carbon allocation for pastures in the crop and land-use version of LPJ-GUESS, we added the management routines grazing, mowing and fertilization with both mineral N and manure on a daily basis. We assume that in a given grid cell, grasslands are either cut or grazed, and do not consider mixed management (mowing and grazing).

Mowing can be triggered either by providing specific harvest dates or is calculated dynamically as a function of LAI, and occurs every 30 days or more. After mowing, the LAI value is decreased to a fixed value of 0.5. Immediately after each of the first three cuts in a year, pastures are fertilized with mineral N and manure. We assume that mineral N fertilizer is distributed equally to the three mowing events (33% each). Manure is only applied after the first cut. 100% of harvest goes to the atmosphere while 10% of biomass not harvested goes to litter (biomass loss during harvest).

Grazing is simulated only at LAI values above 0.5. In case of LAI values below this threshold, the simulated grazing stops. Grazing resumes again when simulated LAI becomes greater than the threshold value. This approach is similar to [24] who used a threshold value of shoot biomass to determine the grazing period. For grazed pastures, a regular, relatively high daily grazing intensity of 2.5% of foliage was assumed. This means pastures are grazed close to their carrying capacity. More extensively grazed grasslands are not simulated in this study but this can easily be done by lowering the grazing intensity. Of each daily feed, 60% of the carbon (C) is assumed to be lost to the atmosphere directly, 15% C is incorporated into the animals body mass while the remaining 25% C return to the paddock in dung and urine (similar to [25, 26]). For N, 75% N is returned to the paddock as part of dung and urine [27–29] while the remaining 25% N is incorporated into the animals body mass. C and N incorporated into the animals bodies is moved to the slow carbon pool with a turnover rate of five years (representing both C and N stored in animal biomass during lifetime). Grazed pastures are fertilized three times per year, every two months with mineral N and manure.

Gridded mineral fertilizer and manure nitrogen application rates for European grasslands in the European Union (EU27) were estimated by the Common Agricultural Policy Regionalized Impact analysis (CAPRI) model (see [30, 31]) based on combined information from official and harmonized data sources such as EUROSTAT [32] and FAOstat [33]. It was spatially dis-aggregated using the methodology described in [34]. The data were estimated at a spatial resolution of 1 km and were re-aggregated here to a spatial resolution of 0.5°. For French regions, more detailed data from the French national statistics were used [35]. We used a set of rules to rebuild the temporal evolution of gridded nitrogen fertilization from 1901 to 2010. First, organic fertilizer was assumed to have remained constant over time (due to the lack of statistical data). Second, the application rate of mineral fertilizer evolved with time following the total mineral nitrogen fertilizer consumption of the European Union [36]. Third, mineral fertilizers were set to be applied since 1951, and application rates linearly increased from 0 to the observed level of 1961 during the period 1951-1960.

Manure is represented by an increase in the metabolic and structural soil organic matter (SOM) pool with a C to N ratio (C:N) of 30. This value has been chosen to represent the C and N content from sources ranging from poultry waste (C:N of ca. 15) to straw-rich manure from livestock (C:N of 40 or more). Since both metabolic and structural SOM pools have different turnover rates, N derived from manure becomes available for an extended period in the soil. Besides nitrogen fertilizer application, nitrogen deposition and nitrogen fixation by soil microorganisms were considered as nitrogen addition as well (see [15]). This means that for the grid cells with no fertilizer application, there were potentially still nitrogen inputs by deposition and fixation.

To account for changes in land-use intensity in the future, we used data from [37] who projected intensive and extensive use of pastures until 2040. The authors used grazing intensity of cattle, goats, and sheep [38] as a proxy for nitrogen inputs on pastures as suggested by [39]. This data were then disaggregated and reclassified into two classes, which were used as a proxy for low and high grassland intensity. More information can be found in [37]. We aggregated this data to 0.5° and assumed an increase in N application of 50 kg/ha (mean N application calculated over Europe) when a gridcell changed from extensive to intensive. We reduced N application by 50 kg/ha for the opposite change. For the missing years, we conducted a linear interpolation.

### 2.2 Calibration, validation & experimental setup

We calibrated the model using LAI measurements from an intensively managed grassland in Oensingen (OEN), located in Switzerland (47°17’N, 07°44’E) at 450 m above sea level, with an annual mean temperature of 9°C and annual precipitation of 1100 mm yr-1. The OEN grassland has been newly sown in spring 2001 with grass and clover. The soil type is stagnic Cambisol (eutric) with a soil organic matter content of 3.5%. It is cut four times a year and fertilizers are applied as solid ammonium nitrate or liquid cattle manure (ca. 200 kg N ha-1 yr-1). Since the N application rates and dates were not reported, we distributed the 200 kg N ha^−1^ yr^−1^ in equal applications after each harvest for the calibration runs. The calibration was conducted for 10000 parameter sets of four parameters relevant for daily C allocation and management: c_pft_ (PFT-specific parameter used for calculating the growth factor), s_tor_ (fraction of the root carbon content), *α* (transfer rate constant) and f_root_ (fraction of roots dying off after a mowing event). Each parameter was sampled from a uniform distribution on the interval from 0.001 to 1. We calculated the log-likelihood for each observed data point *y*_*i*_ (where *i* = 1, …, *n*) under the assumption of a normal distribution, with the corresponding assessment from model simulation *j*, *M*_*ij*_, as mean and a standard deviation *σ*:

$$\begin{array}{c} y_{i}|M_{ij}∼N\left(M_{ij},\;\sigma^{2}\right)\; \end{array}$$ (1)

where *j* = 1: 10000 denotes the different model runs given different parameter sets and *n* is the length of the time series (here 63). Based on the log-likelihood, we then calculated weights for each model run by dividing the sum of the log-likelihood of each run by the overall sum of the log-likelihood:

$$\begin{array}{c} w_{j}=\frac{\sum_{i=1}^{I}loglik\left(y_{i}|M_{ij}\right)}{\sum_{j=1}^{J}\left(\sum_{i=1}^{I}loglik\left(y_{i}|M_{ij}\right)\right)}. \end{array}$$ (2)

These weights were then used to estimate the posterior distribution of the unknown parameters (given observed data) via importance sampling. We also calculated the Willmotts index of agreement, the Nash–Sutcliffe model efficiency coefficient, the root-mean-square error and the Pearson correlation coefficient to assess model-data agreement for the calibration. The Willmotts index of agreement [40] thereby spans between -1 and +1 with values approaching +1 representing better model performance. The Nash–Sutcliffe model efficiency coefficient [41] ranges from -Inf to 1. Essentially, the closer to 1, the more accurate the model is. The root-mean-square error is the standard deviation of the residuals and has the same unit as the dependent variable. Finally, the Pearson correlation coefficient is measure of the linear correlation between observed and simulated values. A coefficient of 1 means a perfect positive correlation while a value of -1 means a perfect negative correlation.

In order to regionally validate the newly extended and calibrated model and assess the spatial pattern of modeled potential (maximum) productivity (defined as annual production of forage from cut grassland), we compared potential productivity with data from [8]. In this study, the authors constructed a map of Europe showing the spatial distribution of grassland productivity by integrating census statistics, literature, and expert judgment using the NUTS (Nomenclature of Territorial Units for Statistics) classification. The data is provided at NUTS-2 to country level, depending on data availability. The biological potential of grassland productivity from LPJ-GUESS (on a spatial resolution of 0.5°) was averaged over the period 1995-2004 and aggregated to the NUTS-2 level weighted by the corresponding grassland area in each grid cell (derived from [42]). Further, we compared our simulated potential productivity to simulations from ORCHIDEE-GM [43]. ORCHIDEE-GM [18] is a version of ORCHIDEE [44] that has been recently developed to explicitly represent grassland management (GM) such as mowing and livestock density. Grassland management was developed by implementing the management module from PaSim [45, 46], a grassland model developed initially for site applications, into ORCHIDEE. For the regional validation, we used the N application data derived from [4] and CAPRI (described above) as well as WATCH climate data [47]. The simulations (starting 1901) were thereby spun up for 500 model years in order to achieve equilibrium in carbon pool sizes with respect to the long-term climate. The soil carbon pool size was solved analytically during spin-up in order to reduce computation time.

For the sake of testing the implications of including land-use intensity (LUI) for pastures, we selected three variables: net primary productivity (NPP), net ecosystem carbon balance (NECB) and nitrogen leaching. NECB thereby reflects not only photosynthesis and heterotrophic respiration but also other carbon transfers including losses by harvest. In this study, it is negative in case of C sinks and positive in case of C sources. However, carbon from fossil fuels used in the manufacture of fertilizer and the use of farmland equipment is not taken into account here. We conducted ecosystem simulations which included not only pastures, but also croplands and managed forests for geographical Europe with the Ural Mountains as border in the east and the Caucasus Mountains as borders in the south-east. On croplands, only maize and wheat as the dominant crops were simulated. For the NUTS-2 regions, historical N applications for 1990-2008 were used which were estimated by the CAPRI model based on combined information from official and harmonized data sources. For the remaining regions, we used N application data derived from [4] which consist of long-term mean N fertilizer centered on the year 2000 for maize and wheat on a national and subnational level. Historical N deposition data was taken from [48] as estimated with the CAM model [49]. For pastures, we used the N fertilizer data described above (data from CAPRI and [4]). We conducted two runs with mowing and grazing only and mixed them as a post process assuming a fraction of 50% each.

**Fig 2:**
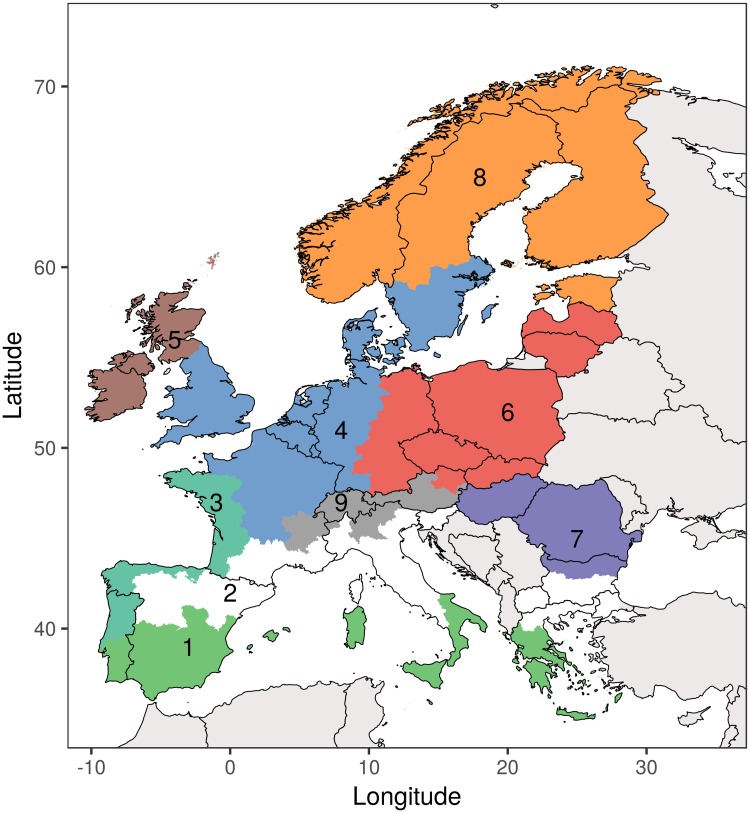
Agro-climatic zones used for zonal statistics in the analysis. 1 = Mediterranean South; 2 = Mediterranean North; 3 = Atlantic South; 4 = Atlantic Central; 5 = Atlantic North; 6 = Continental North; 7 = Continental South; 8 = Boreal; 9 = Alpine.

For the future climate simulations (ending 2100), monthly climate data from six General Circulation Models (GCMs) taking part in CMIP5 (Coupled Model Intercomparison Project Phase 5) were selected: CCSM4 (Community Climate System Model version 4, [50]), MPI-ESM-LR (Max Planck Institute Earth System Model, [51]), IPSL-CM5A-LR (Institut Pierre Simon Laplace coupled model version 5A, [52]), NORESM (Norwegian Earth System Model version 1, [53], GFDL-CM3 (Geophysical Fluid Dynamics Laboratory Coupled Physical Model version 3, [54], GISS-E2-R (Goddard Institute for Space Studies ModelE2, [55]). The GCM climate data cover the period between 1850 and 2100 and were bias corrected against Climatic Research Unit (CRU) data [56] for monthly means over the period from 1961 to 1990, as described by [57]. In order to show future trajectories for potential grassland productivity, we used the agro-climatic regions as defined by [58] to calculate the zonal mean for the time series 2010 to 2050 (see Fig 2).

## 3 Results

### 3.1 Calibration & regional validation

**Fig 3:**
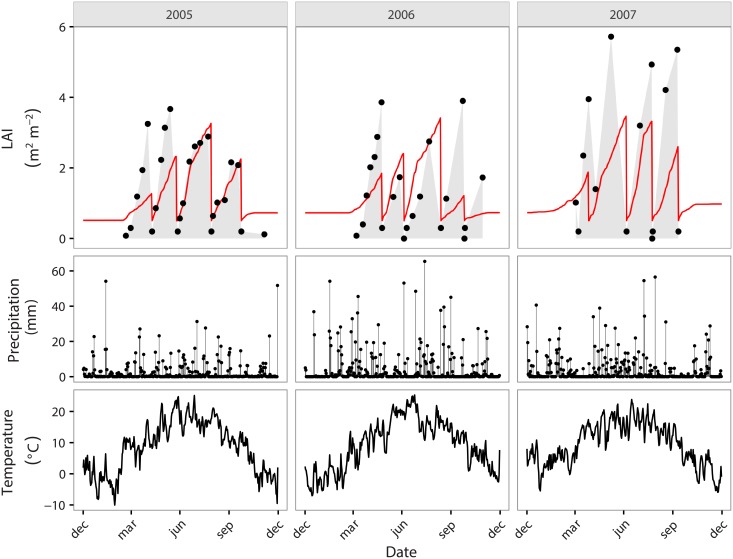
Comparison of simulated (red line) and observed (black dots) leaf area index (LAI) for the cut grassland of Oensingen (CH-OEN) after calibration. Measured precipitation and temperature are given as reference.

Fig 3 shows the comparison of simulated and observed LAI for OEN over a period of three years after calibration. The observed LAI values in this period drop abruptly immediately after mowing and restore to high values of about 3-4 *m*^2^*m*^−2^ within a relatively short time period (ca. two weeks) after mowing.

Overall, the calibrated model follows the observations quite well, especially in the middle of the growing season. However, LPJ-GUESS-LUI tends to underestimate the first harvest and cannot reproduce the steep LAI increase observed in early spring. Additionally, the comparatively high LAI in 2007 are not captured either. This observed increased LAI is most likely not generated by climate since there is no significant increase in temperature, precipitation amount and number of rain days evident (see Fig 3). The mean temperature of 2007 is with 9.6°C only slightly higher than in 2006 (9.3°C). Mean precipitation (3.7 mm) in 2007 is less than in 2006 (4.3 mm) and number of rain days (188 days) is equal to 2006 (185 days) and substantially less than in 2005 (218 days). The following model evaluation metrics were calculated over the three years of data: Willmotts index of agreement: 0.70; Nash–Sutcliffe model efficiency coefficient: 0.45; root-mean-square error: 1.1 *m*^2^*m*^−2^; Pearson correlation coefficient: 0.76; mean bias: -0.37 *m*^2^*m*^−2^.

**Fig 4:**
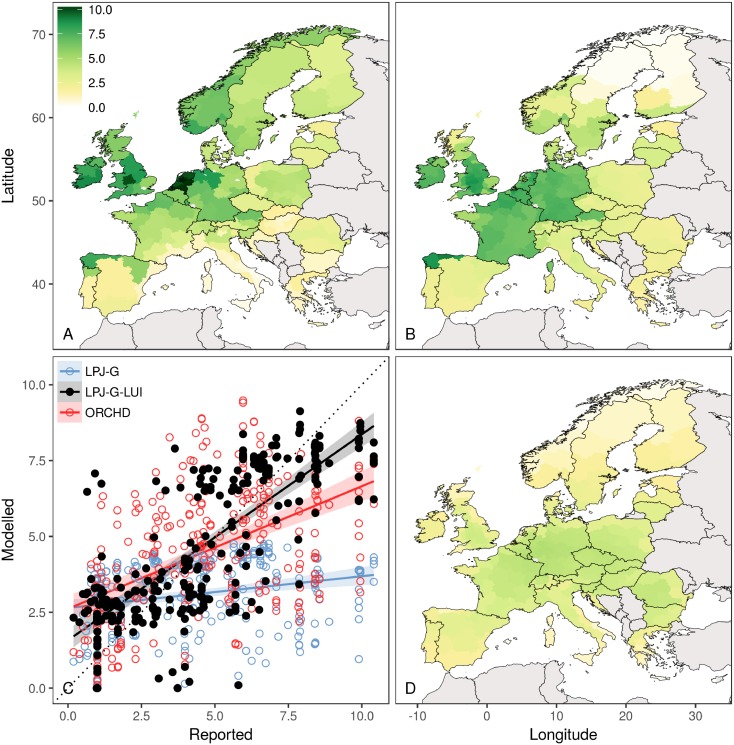
Regional validation at NUTS-2 level. Spatial distribution of (A): actual grassland forage productivity from [8], (B): potential grassland productivity from cut grasslands simulated by the updated LPJ-GUESS version which includes the representation of land-use intensity, (D): potential grassland productivity simulated by the default LPJ-GUESS version without detailed management regimes. (C): Scatterplot of productivity reported from [8] versus simulated data from the default version of LPJ-GUESS (blue), the updated version LPJ-GUESS-LUI (black) and simulated data with ORCHIDEE-GM (red) from [43]. The dotted line in the scatter plot indicates the 1:1 line.

When the simulated productivity at the pixel level is aggregated over the EUROSTAT administrative regions, a significant improvement in fit is obtained between simulated and reported productivity across the 272 regions (see Fig 4). While the standard pasture representation described by [14] results in a rather homogeneous pattern stretching over France, Germany and eastern Europe, LPJ-GUESS-LUI lines out the hotspots of high productivity in central Europe, mostly the Netherlands, Germany and France. However, LPJ-GUESS-LUI tends to simulate a higher potential productivity than the actual productivity (yield) reported in [8] for the Mediterranean such as Spain, Italy and Greece. This result is logical because the model simulates the potential (maximum) productivity of permanent cut grassland, whereas the reported productivity is based on actual harvest data. Exceptions are northern Spain, Norway, and northern Sweden where LPJ-GUESS-LUI simulated lower productivity than reported from national statistics. The scatterplot (see Fig 4C) clearly demonstrates that the potential productivity simulations are now more in range with the observations while LPJ-GUESS mostly underestimates the productivity. Thereby, the spread in simulated output of LPJ-GUESS-LUI on either site of the 1:1 line is in range with simulations from ORCHIDEE-GM [43].

### 3.2 Implications for C and N fluxes

In order to provide some sort of sensitivity estimations of LPJ-GUESS to the pasture management modifications and outline the implications for continental Europe, we simulated pastures, croplands and managed forests. Each land cover type was simulated independently while land cover fractions were used for weighing the simulation results to provide one combined estimate for Europe. It can be expected that the changes to the model structure have few effects for gridcells that have small landcover fractions of pastures since cropland and forest management were not altered between the two LPJ-GUESS versions.

**Fig 5:**
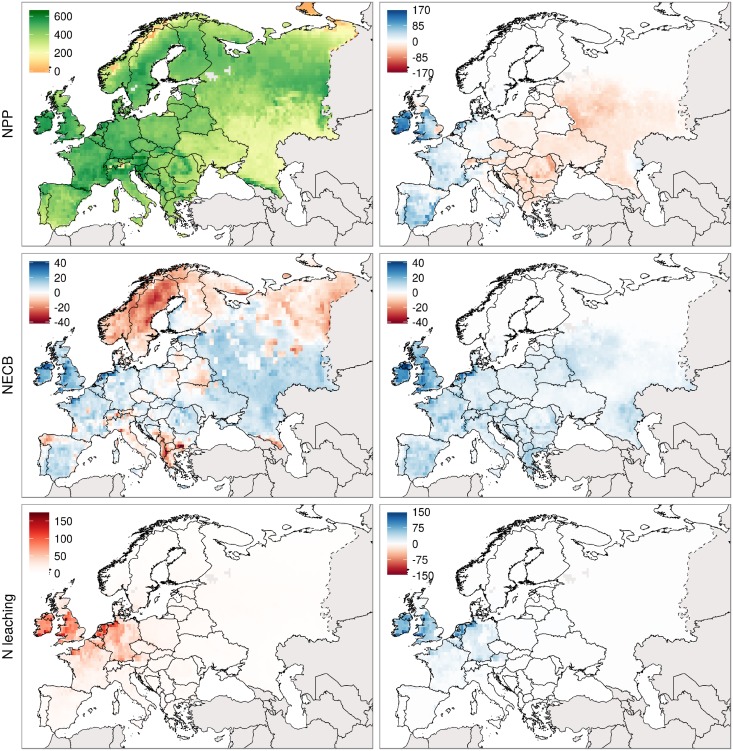
Simulated NPP, NECB and NENB from managed forests, croplands and pastures. The first map in each row displays average simulations for 1995-2004 as calculated with the improved version while the second column displays the difference between improved version and default version of LPJ-GUESS.

Fig 5 shows simulations of NPP, NECB and N leaching from managed forests, croplands and pastures (in each row). The left panel displays average simulations for 2001-2010 as calculated with LPJ-GUESS-LUI while the right panel displays the differences between LPJ-GUESS-LUI and LPJ-GUESS. NPP reaches values of 600 gC m^−2^ in the mountainous and moist regions of central Europe such as the Massif Central in southern France, the Alps and the Carpathian Mountains in Romania. Very low values can be found in the cold regions of Norway and Siberia while low to medium values are estimated for the cool and dry regions north of the Caucasus mountains in Russia and Ukraine. The comparison of the improved version of LPJ-GUESS with the standard version points out the implications of including both daily carbon allocation for pastures and land-use intensity data in form of fertilizer and manure into LPJ-GUESS. This affected the simulations in two ways. NPP increased in the range from 50 to 160 gC m^−2^ in large parts of the Mediterranean, the Atlantic Central and the Atlantic North such as Spain, France, the Netherlands, Germany and Ireland. However, NPP decreased by about 50 gC m^−2^ in eastern Europe, mainly Russia and Romania. The combined NECB from forests, croplands and pastures displays two opposing patterns across Europe. While there is an uptake of carbon of up to 40 gC m^−2^ in large parts of the Boreal, northern Russia and parts of the Mediterranean (Italy and Greece), there is a source of carbon of about 20 gC m^−2^ in the rest of the continent, including the Iberian Peninsula, France, Germany, the United Kingdom and eastern Europe such as the southern parts of Russia. The comparison between LPJ-GUESS-LUI and the standard version of LPJ-GUESS reveals that NECB is uniformly increased by up to 20 gC m^−2^ in areas with high pasture fractions, particularly in central France, the United Kingdom and northern Germany. Combined N leaching from croplands and pastures is highest in the Atlantic Central and western parts of the Continental North. The highest values can be found in the Netherlands and Germany with N leaching of up to 90 kg N ha^−1^. Including pasture land-use intensity into LPJ-GUESS increased leaching in mainly in northern Germany and the Netherlands by up to 60 kg N ha^−1^.

### 3.3 Future scenario experiment

**Fig 6:**
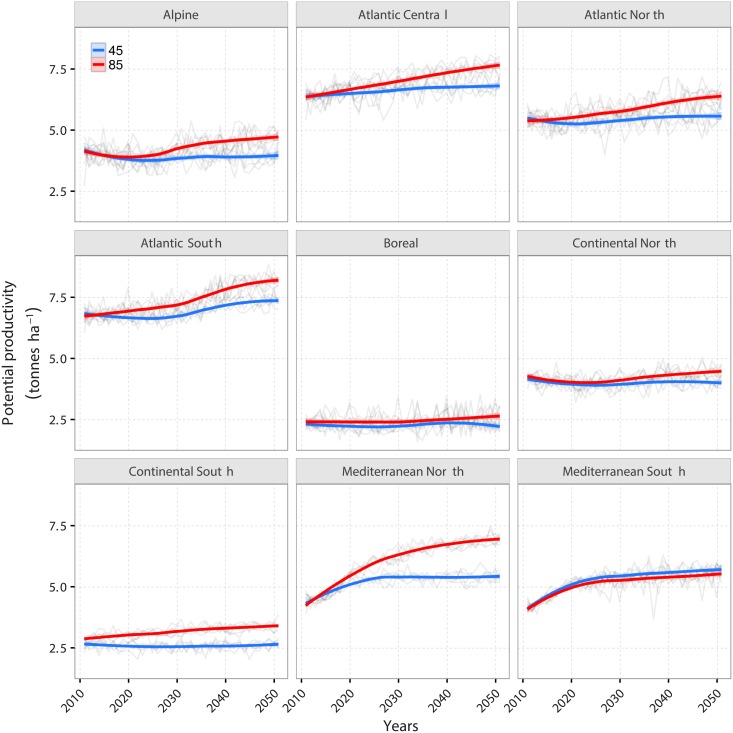
Future trajectory of potential grassland productivity for nine agro-climatic regions between 2010 and 2050. The results for the individual GCMs and RCPs are given as black lines while the red and blue line indicates the locally weighted regression (loess) averaged over the ensemble for the two RCPs.

The future scenario experiment in which we forced LPJ-GUESS-LUI with climate data from six different GCMs under two RCPs, changes in CO_2_ concentration, nitrogen deposition and future land-use intensity data reveals different trends (see Fig 6) for the specific agro-climatic regions (see Fig 2).

In the Boreal and the Continental North, LPJ-GUESS simulated a rather constant potential grassland productivity between 2010 and 2050. In the Continental South, the Alpine and the Atlantic North, there is a constant productivity for RCP 4.5 while there is a slight increase in productivity under RCP 8.5. In the Atlantic Central and the Atlantic South, productivity increases under both RCPs, with productivity crossing 7.5 tonnes ha^−1^ under RCP 8.5. The pattern for both Mediterranean North and Mediterranean South are distinctly different to the other agro-climatic regions. Here, productivity increases rather steep until about 2020 and then starts leveling off. Overall, the Mediterranean North is the only area where under both emission trajectories, LPJ-GUESS simulates a clearly diverging potential productivity. While in 2050, up to 7 tonnes ha^−1^ are projected under RCP 8.5, productivity under RCP 4.5 does not reach 5.5 tonnes ha^−1^. Overall, the variability between GCMs and RCPs is overlapping for most regions, demonstrating a limited importance of the representative concentrations pathways.

**Fig 7:**
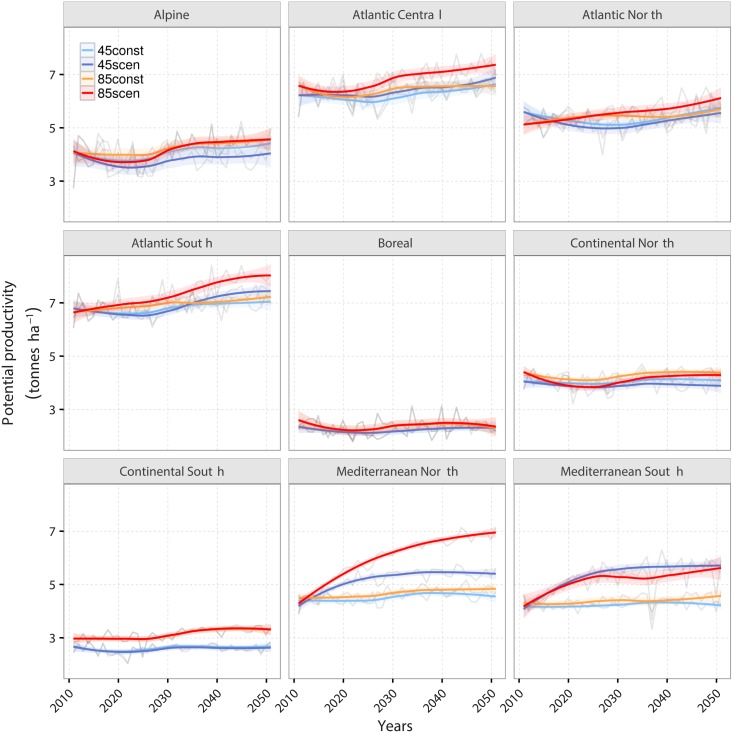
Future trajectory of potential grassland productivity for nine agro-climatic regions between 2010 and 2050 for four simulations. The light blue line (45const) indicates the locally weighted regression (loess) averaged over the simulations under RCP 4.5 conducted with a constant N application rate. The dark blue line (45scen) displays the loess of simulations under the same RCP with scenario N application rates derived from [37]. The orange line (85const) indicates the loess under RCP 8.5 with constant N applications while the red line (85scen) displays the loess under the same RCP with scenario N application rates derived from [37].

By keeping the N application data constant (45const and 85const in Fig 7), it becomes clear, that the strong increase in productivity in the Mediterranean North and South are caused by the increases in N application. Without changes in land-use intensity, productivity increases only slightly in the Mediterranean North while it stays constant in the Mediterranean South. Also the productivity increases in the Atlantic Central and Atlantic South under RCP 8.5 can be explained by changes in land-use intensity mostly.

## 4 Discussion

In the present study, we simulate and analyze the implications of accounting for land-use intensity in the form of N fertilizer and manure and daily pasture management on spatial and temporal patterns of European grassland processes.

The results of this study demonstrate that potential grassland productivity cannot be adequately simulated by only driving the model with climate and CO_2_ while ignoring land-use intensity. However, LPJ-GUESS-LUI simulations which are based on daily carbon allocation for grasses, land-use intensity data and daily management of pastures prove to strongly improve the representation of LAI site observations and regionally reported data on grassland productivity. The site validation shows that calibrated LPJ-GUESS-LUI simulations follow the temporal observations quite well, especially in the middle of the growing season. However, the model seems to consistently underestimate the steep increase of LAI in spring while it captures growth in the second half of the year. The reason for this might be that the root storage for the early boost in spring is not adequately simulated since LAI is only reduced to 0.5. The increased LAI in 2007 is not captured either that year. However, neither mean temperature, nor mean precipitation and number of rain days can explain the increased amount of leaf material. This indicates that a process other than climate, that we do not account for in our approach, might be responsible for the increase (e.g. management). This is partially supported by the fact that ORCHIDEE-GM does not simulate this increased response either [18]. The regional validation shows that the spatial pattern of grassland productivity are improved compared to the standard version of LPJ-GUESS. The grassland productivity simulated by LPJ-GUESS-LUI represents thereby a potential estimate (in equilibrium with e.g. climate and CO_2_ concentration), but still neglects site-specific properties such as terrain, slope, grass species and nutrient availability other than N from fertilization, deposition and microbial fixation. The reported data from [8] includes actual production (partly estimated from expert judgments) in each region under different types of management, which is often restricted by limiting soil nutrients and low herbage quality. One example of an overestimation is the simulated productivity in southern France (Fig 4) which has also been observed for ORCHIDEE-GM [43]. This is possibly due to the model’s missing representation of soil nutrients as well as to the low aboveground productivity of specific grass species used in this area. Detailed spatial data on e.g. soil fertility might improve the model´s capacity to simulate grassland productivity. Nevertheless, the spatial distribution of modeled potential productivity agrees well with statistics (Fig 4). LPJ-GUESS-LUI realistically reproduces high productivity in humid and oceanic regions and low productivity in dry regions with the Mediterranean climate affected by drought. The standard version of LPJ-GUESS is not able to simulate the regions of high productivity such as UK, the Netherlands and Germany. This confirms the combined role played by both water limitation and nitrogen limitation in the productivity of managed grassland ecosystems.

As shown by Fig 5, accounting for pasture fertilization intensity and management regimes had three main overall effects on the performance and behavior of LPJ-GUESS. First, NPP in western Europe, NECB and N leaching are increased. The increase in NPP and N leaching can be explained by the high amounts of fertilization in western Europe. The overall increase in NECB is instead more influenced by the (relatively intensive) management regimes that in turn led to e.g. high harvest fluxes. Second, NPP is reduced in eastern and south-eastern Europe compared to the default version of LPJ-GUESS. In these regions, N fertilization as reported by the CAPRI model (for EU27) and [4] is very low or zero while the harvesting and grazing intensity is still comparatively high. This leads to a depletion of the soil and an overuse of the pastures so that a high productivity cannot be sustained. One possibility to avoid the overuse in these parts of Europe is to use further land-use intensity data such as grazing intensity and number of harvests per year. While there is data to quantify global grazing intensity [59, 60], data on the number of harvests is nonexistent on a continental or global scale. Third, accounting for daily management also brings the opportunity to simulate harvest of pastures at specific harvest dates which was not possible in previous modeling studies even though such data was available (e.g. [61]).

The simulated future increase in grassland productivity for large regions in Europe has also been shown by [62]. In their study, grassland productivity was simulated to increase with further warming, mainly due to rising concentrations of CO_2_. However, compared to our study, they found a decrease in productivity in the Atlantic North for which our simulations showed an increase under RCP 8.5 and no trend for RCP 4.5. Productivity is also suggested to increase for grassland ecosystems beyond Europe. [3] projected widespread and consistent increases in vegetation fractional cover (until 2100) throughout most of North America, despite the projected increase in aridity.

Our study has demonstrated the importance of including land-use intensity data in form of N applications into simulations with DGVMs for global change studies. While there are some data sets on current and historical N applications for Europe (e.g. derived from CAPRI) and the world [4], there is a strong need for more detailed data on a finer spatial resolution since many regions are currently only represented on a national or subnational level. In general, the current status of data availability is intermediate. Existing datasets are often model derived and characterized by large gaps and uncertainties, particularly relating to spatial patterns and livestock manure [63]. While fertilization scenario data exists for specific crop types [37, 64], more efforts need to be done for managed grasslands given their high importance in the landscape of Europe. Some attempts have been made by [65] which were further processed and used by [37]. However, the projections developed in the latter study contain only the two classes “intensive” and “extensive” which is insufficient for further detailed use in ecosystem models. The importance of N application projections for crops has been recently stressed by [11]. Since the pasture area is projected to decrease in Europe [12] accompanied with an intensification of livestock production systems, changes in land-use intensity for grasslands can be expected as well. Although our work confirms that climate change and CO_2_ can affect many ecosystem-based functional attributes, it also suggests that land management practices remain the dominant drivers in determining the performance of grassland plant communities. Land management may thus be critical for influencing projected responses to future climate change and elevated CO_2_ in models of grassland function at least for factors relating to primary production [5].

The incorporation of daily management regimes and N fertilization data does not come without limitations. One of the benefits over other studies [43] is certainly that we have a full N cycle implemented in the model and force the model with both N deposition and pasture fertilization data. The added management routines are kept simple and generic to allow global simulations while keeping the computational burden low. Trampling by livestock is not taken into account but can potentially be added via e.g. decreasing biomass and inclusion of livestock density data from [59]. A limitation of our study is also that a large proportion of the labile forms of N present in livestock excreta is unable to be taken up by plants and end up being either volatilized or denitrified [27]. For this study, mowing and grazing runs were mixed with 50% each. This is rather unrealistic, but spatial data is lacking since existing national and gridded data sets do not separate grazing and mowing. There is however a potential for future studies to use remote sensing and mowing detecting algorithms to fill that knowledge gap [66]. Another possibility is to optimize the grazing and mowing ratios similar to the PaSim model [24]. Finally, our study was conducted at a spatial resolution of 0.5° which will have an effect mostly on the mountainous regions in which a finer resolution would improve the result [67].

## 5 Conclusion

The standard pasture representation of LPJ-GUESS which is mainly driven by climate variables, CO_2_ concentration and nitrogen deposition is not able to reproduce the spatial patterns of potential grassland productivity in Europe. The extended version LPJ-GUESS-LUI which includes daily carbon allocation, land-use intensity in form of N fertilizer and manure and daily management regimes instead shows an improved fit to reported data. This stresses the importance of management variables over climate variables in the context of managed grassland productivity. We conclude that even though the current status of data availability is intermediate, it is important to advance with incorporating grassland management intensity in form of N fertilization in dynamic vegetation models. Incorporating land-use intensity will improve predictions of terrestrial C sinks and sources as well as N leaching.

## References

[1] Corrall A. Prediction of production from grassland. Information Bulletin of the FAO European Research Co-operative Network on Pastures and Field Crops, Herba. 1988;1:25–28.

[2] Schulze ED. Carbon and Nitrogen Cycling in European Forest Ecosystems. New York: Springer; 2000.

[3] Hufkens K, Keenan TF, Flanagan LB, Scott RL, Bernacchi CJ, Joo E, et al Productivity of North American grasslands is increased under future climate scenarios despite rising aridity. Nature Climate Change. 2016;6 10.1038/nclimate2942

[4] Mueller ND, Gerber JS, Johnston M, Ray DK, Ramankutty N, Foley JA. Closing yield gaps through nutrient and water management. Nature. 2012;490(7419):254–257. 10.1038/nature11420 22932270

[5] Thébault A, Mariotte P, Lortie CJ, MacDougall AS. Land management trumps the effects of climate change and elevated CO_2_ on grassland functioning. Journal of Ecology. 2014;102(4):896–904. 10.1111/1365-2745.12236

[6] Levers C, Verkerk PJ, Müller D, Verburg PH, Butsic V, Leitão PJ, et al Drivers of forest harvesting intensity patterns in Europe. Forest Ecology and Management. 2014 3;315:160–172. 10.1016/j.foreco.2013.12.030

[7] Levers C, Butsic V, Verburg PH, Müller D, Kuemmerle T. Drivers of changes in agricultural intensity in Europe. Land Use Policy. 2016;58:380–393. 10.1016/j.landusepol.2016.08.013

[8] Smit HJ, Metzger MJ, Ewert F. Spatial distribution of grassland productivity and land use in Europe. Agricultural Systems. 2008;98(3):208–219. 10.1016/j.agsy.2008.07.004

[9] Foley Ja, Monfreda C, Ramankutty N, Zaks D. Our share of the planetary pie. Proceedings of the National Academy of Sciences of the United States of America. 2007 7;104(31):12585–6. 10.1073/pnas.0705190104 17646656 PMC1937509

[10] Asselen S, Verburg PH. A Land System representation for global assessments and land-use modeling. Global Change Biology. 2012 10;18(10):3125–3148. 10.1111/j.1365-2486.2012.02759.x 28741836

[11] Webber H, Zhao G, Wolf J, Britz W, de Vries W, Gaiser T, et al Climate change impacts on European crop yields: Do we need to consider nitrogen limitation? European Journal of Agronomy. 2015;71:123–134. 10.1016/j.eja.2015.09.002

[12] Lehsten V, Sykes MT, Scott AV, Tzanopoulos J, Kallimanis A, Mazaris A, et al Disentangling the effects of land-use change, climate and CO_2_ on projected future European habitat types. Global Ecology and Biogeography. 2015; p. n/a–n/a. 10.1111/geb.12291

[13] Prentice IC, Bondeau A, Cramer W, Harrison SP, Hickler T, Lucht W, et al In: Canadell JG, Pataki DE, Pitelka LF, editors. Dynamic Global Vegetation Modeling: Quantifying Terrestrial Ecosystem Responses to Large-Scale Environmental Change. Berlin, Heidelberg: Springer Berlin Heidelberg; 2007 p. 175–192.

[14] Lindeskog M, Arneth A, Bondeau A, Waha K, Seaquist J, Olin S, et al Implications of accounting for land use in simulations of ecosystem carbon cycling in Africa. Earth System Dynamics. 2013;4:385–407. 10.5194/esd-4-385-2013

[15] Smith B, Wårlind D, Arneth A, Hickler T, Leadley P, Siltberg J, et al Implications of incorporating N cycling and N limitations on primary production in an individual-based dynamic vegetation model. Biogeosciences. 2014;11(7):2027–2054. 10.5194/bg-11-2027-2014

[16] Smith B, Prentice IC, Sykes MT. Representation of vegetation dynamics in the modelling of terrestrial ecosystems: comparing two contrasting approaches within European climate space. Global Ecology and Biogeography. 2001 6;10(6):621–637. 10.1046/j.1466-822X.2001.t01-1-00256.x

[17] Olin S, Schurgers G, Lindeskog M, Wårlind D, Smith B, Bodin P, et al Modelling the response of yields and tissue C: N to changes in atmospheric CO_2_ and N management in the main wheat regions of western Europe. Biogeosciences. 2015;12(8):2489–2515. 10.5194/bg-12-2489-2015

[18] Chang JF, Viovy N, Vuichard N, Ciais P, Wang T, Cozic A, et al Incorporating grassland management in ORCHIDEE: model description and evaluation at 11 eddy-covariance sites in Europe. Geoscientific Model Development. 2013;6(6):2165–2181. 10.5194/gmd-6-2165-2013

[19] Parton WJ, Scurlock JMO, Ojima DS, Gilmanov TG, Scholes RJ, Schimel DS, et al Observations and modeling of biomass and soil organic matter dynamics for the grassland biome worldwide. Global Biogeochemical Cycles. 1993;7(4):785–809. 10.1029/93GB02042

[20] Bondeau A, Smith PC, Zaehle S, Schaphoff S, Lucht W, Cramer W, et al Modelling the role of agriculture for the 20th century global terrestrial carbon balance. Global Change Biology. 2007 3;13(3):679–706. 10.1111/j.1365-2486.2006.01305.x

[21] Waha K, van Bussel LGJ, Müller C, Bondeau A. Climate-driven simulation of global crop sowing dates. Global Ecology and Biogeography. 2012;21(2):247–259. 10.1111/j.1466-8238.2011.00678.x

[22] Boke-Olén N, Lehsten V, Abdi AM, Ardö J, Khatir AA. Estimating grazing potentials in Sudan using daily carbon allocation in a dynamic vegetation model. Journal of arid environments; under review.

[23] Johnson IR, Thornley JHM. Vegetative crop growth model incorporating leaf area expansion and senescence, and applied to grass. Plant, Cell & Environment. 1983;6(9):721–729.

[24] Vuichard N, Ciais P, Viovy N, Calanca P, Soussana JF. Estimating the greenhouse gas fluxes of European grasslands with a process-based model: 2. Simulations at the continental level. Global Biogeochemical Cycles. 2007;21(1):1–13. 10.1029/2005GB002612

[25] Rolinski S, Weindl I, Heinke J, Bodirsky BL, Biewald A, Lotze-Campen H. Pasture harvest, carbon sequestration and feeding potentials under different grazing intensities. Advances in Animal Biosciences. 2015;6:43–45. 10.1017/S2040470014000521

[26] Kirschbaum MUF, Rutledge S, Kuijper IA, Mudge PL, Puche N, Wall AM, et al Modelling carbon and water exchange of a grazed pasture in New Zealand constrained by eddy covariance measurements. Science of the Total Environment. 2015;512-513:273–286. 10.1016/j.scitotenv.2015.01.045 25634732

[27] Whitehead DC. Grassland Nitrogen. Wallingford: CAB International; 1995.

[28] Petersen SO, Sommer SG, Aaes O, Søegaard K. Ammonia losses from urine and dung of grazing cattle: effect of N intake. Atmospheric Environment. 1998;32(3):295—300. 10.1016/S1352-2310(97)00043-5

[29] Sannes RA, Messman MA, Vagnoni DB. Form of Rumen-Degradable Carbohydrate and Nitrogen on Microbial Protein Synthesis and Protein Efficiency of Dairy Cows1. Journal of Dairy Science. 2002;85(4):900—908. 10.3168/jds.S0022-0302(02)74148-9 12018435

[30] Britz W, Witzke H. CAPRI model documentation 2012. Available at: www.capri-model.org. 2012;.

[31] Leip A, Britz W, Weiss F, De Vries W. Farm, land, and soil nitrogen budgets for agriculture in Europe calculated with CAPRI. Environmental Pollution. 2011;159(11):3243–3253. 10.1016/j.envpol.2011.01.040 21420769

[32] EUROSTAT Agri-environmental indicators;. http://ec.europa.eu/eurostat/web/agri-environmental-indicators/indicators.

[33] FAOstat;. http://faostat3.fao.org.

[34] Leip A, Marchi G, Koeble R, Kempen M, Britz W, Li C. Linking an economic model for European agriculture with a mechanistic model to estimate nitrogen and carbon losses from arable soils in Europe. Biogeosciences. 2008;5(1):73–94. 10.5194/bg-5-73-2008

[35] AGRESTE statistics;. http://agreste.agriculture.gouv.fr.

[36] Tenkorang F, Lowenberg-Deboer J. Forecasting long-term global fertilizer demand. Nutrient Cycling in Agroecosystems. 2009;83(3):233–247. 10.1007/s10705-008-9214-y

[37] Stürck J, Levers C, van der Zanden EH, Schulp CJE, Verkerk PJ, Kuemmerle T, et al Simulating and delineating future land change trajectories across Europe. Regional Environmental Change. 2015;p. 1–17.

[38] Neumann K, Elbersen BS, Verburg PH, Staritsky I, Pérez-Soba M, de Vries W, et al Modelling the spatial distribution of livestock in Europe. Landscape Ecology. 2009;24(9):1207–1222. 10.1007/s10980-009-9357-5

[39] Temme AJAM, Verburg PH. Mapping and modelling of changes in agricultural intensity in Europe. Agriculture, Ecosystems & Environment. 2011 1;140(1-2):46–56. 10.1016/j.agee.2010.11.010

[40] Willmott CJ, Robeson SM, Matsuura K. A refined index of model performance. International Journal of Climatology. 2012;32(13):2088–2094. 10.1002/joc.2419

[41] Nash JE, Sutcliffe JV. River flow forecasting through conceptual models part I—A discussion of principles. Journal of Hydrology. 1970;10(3):282—290. 10.1016/0022-1694(70)90255-6

[42] Hurtt GC, Chini LP, Frolking S, Betts RA, Feddema J, Fischer G, et al Harmonization of land-use scenarios for the period 1500-2100: 600 years of global gridded annual land-use transitions, wood harvest, and resulting secondary lands. Climatic Change. 2011;109(1):117 10.1007/s10584-011-0153-2

[43] Chang J, Viovy N, Vuichard N, Ciais P, Campioli M, Klumpp K, et al Modeled Changes in Potential Grassland Productivity and in Grass-Fed Ruminant Livestock Density in Europe over 1961–2010. Plos One. 2015;10(5):e0127554 10.1371/journal.pone.0127554 26018186 PMC4446363

[44] Krinner G, Viovy N, de Noblet-Ducoudré N, Ogée J, Polcher J, Friedlingstein P, et al A dynamic global vegetation model for studies of the coupled atmosphere-biosphere system. Global Biogeochemical Cycles. 2005;19(1):n/a–n/a. GB1015. 10.1029/2003GB002199

[45] Riedo M, Grub A, Rosset M, Fuhrer J. A pasture simulation model for dry matter production, and fluxes of carbon, nitrogen, water and energy. Ecological Modelling. 1998;105(2–3):141—183. 10.1016/S0304-3800(97)00110-5

[46] Vuichard N, Soussana JF, Ciais P, Viovy N, Ammann C, Calanca P, et al Estimating the greenhouse gas fluxes of European grasslands with a process-based model: 1. Model evaluation from in situ measurements. Global Biogeochemical Cycles. 2007;21(1):1–14. 10.1029/2005GB002612

[47] Weedon GP, Gomes S, Viterbo P, Shuttleworth WJ, Blyth E, Österle H, et al Creation of the WATCH Forcing Data and Its Use to Assess Global and Regional Reference Crop Evaporation over Land during the Twentieth Century. Journal of Hydrometeorology. 2011;12(5):823–848. 10.1175/2011JHM1369.1

[48] Lamarque JF, Bond TC, Eyring V, Granier C, Heil A, Klimont Z, et al Historical (1850–2000) gridded anthropogenic and biomass burning emissions of reactive gases and aerosols: methodology and application. Atmospheric Chemistry and Physics. 2010;10(15):7017–7039. 10.5194/acp-10-7017-2010

[49] Gent PR, Yeager SG, Neale RB, Levis S, Bailey DA. Improvements in a half degree atmosphere/land version of the CCSM. Climate Dynamics. 2010;34(6):819–833. 10.1007/s00382-009-0614-8

[50] Gent PR, Danabasoglu G, Donner LJ, Holland MM, Hunke EC, Jayne SR, et al The Community Climate System Model Version 4. Journal of Climate. 2011;24(19):4973–4991. 10.1175/2011JCLI4083.1

[51] Stevens B, Giorgetta M, Esch M, Mauritsen T, Crueger T, Rast S, et al Atmospheric component of the MPI-M Earth System Model: ECHAM6. Journal of Advances in Modeling Earth Systems. 2013;5(2):146–172. 10.1002/jame.20015

[52] Dufresne JL, Foujols MA, Denvil S, Caubel A, Marti O, Aumont O, et al Climate change projections using the IPSL-CM5 Earth System Model: from CMIP3 to CMIP5. Climate Dynamics. 2013;40(9):2123–2165. 10.1007/s00382-012-1636-1

[53] Bentsen M, Bethke I, Debernard JB, Iversen T, Kirkevåg A, Seland Ø, et al The Norwegian Earth System Model, NorESM1-M—Part 1: Description and basic evaluation of the physical climate. Geoscientific Model Development. 2013;6(3):687–720. 10.5194/gmd-6-687-2013

[54] Griffies SM, Winton M, Donner LJ, Horowitz LW, Downes SM, Farneti R, et al The GFDL CM3 Coupled Climate Model: Characteristics of the Ocean and Sea Ice Simulations. Journal of Climate. 2011;24(13):3520–3544. 10.1175/2011JCLI3964.1

[55] Schmidt GA, Kelley M, Nazarenko L, Ruedy R, Russell GL, Aleinov I, et al Configuration and assessment of the GISS ModelE2 contributions to the CMIP5 archive. Journal of Advances in Modeling Earth Systems. 2014;6(1):141–184. 10.1002/2013MS000265

[56] Mitchell TD, Jones PD. An improved method of constructing a database of monthly climate observations and associated high-resolution grids. International Journal of Climatology. 2005 5;25(6):693–712. 10.1002/joc.1181

[57] Ahlström A, Smith B, Lindström J, Rummukainen M, Uvo CB. GCM characteristics explain the majority of uncertainty in projected 21st century terrestrial ecosystem carbon balance. Biogeosciences. 2013 3;10(3):1517–1528. 10.5194/bg-10-1517-2013

[58] Iglesias A, Garrote L, Quiroga S, Moneo M. A regional comparison of the effects of climate change on agricultural crops in Europe. Climatic Change. 2012;112:29–46. 10.1007/s10584-011-0338-8

[59] Wint W, Robinson T. Gridded livestock of the world 2007. Washington, D.C: Food and Agriculture Organization of the United Nations; 2007. 20422554

[60] Herrero M, Havlík P, Valin H, Notenbaert A, Rufino MC, Thornton PK, et al Biomass use, production, feed efficiencies, and greenhouse gas emissions from global livestock systems. Proceedings of the National Academy of Sciences of the United States of America. 2013;110(52):20888–93. 10.1073/pnas.1308149110 24344273 PMC3876224

[61] Lautenbach S, Jungandreas A, Blanke J, Lehsten V, Mühlner S, Kühn I, et al Trade-offs between plant species richness and carbon storage in the context of afforestation—Examples from afforestation scenarios in the Mulde Basin, Germany. Ecological Indicators. 2017;73:139–155. 10.1016/j.ecolind.2016.09.035

[62] Chang J, Ciais P, Viovy N, Soussana JF, Klumpp K, Sultan B. Future productivity and phenology changes in European grasslands for different warming levels: implications for grassland management and carbon balance. Carbon Balance and Management. 2017;12(1):11 10.1186/s13021-017-0079-8 28474332 PMC5418182

[63] Erb KH, Luyssaert S, Meyfroidt P, Pongratz J, Don A, Kloster S, et al Land management: data availability and process understanding for global change studies. Global Change Biology. 2016;(4):n/a–n/a. 10.1111/gcb.13443 27447350

[64] Stocker B, Roth R, Joos F. Multiple greenhouse-gas feedbacks from the land biosphere under future climate change scenarios. Nature Climate Change. 2013;3(7):666–672. 10.1038/nclimate1864

[65] Neumann K, Verburg PH, Elbersen B, Stehfest E, Woltjer GB. Multi-scale scenarios of spatial-temporal dynamics in the European livestock sector. Agriculture, Ecosystems and Environment. 2011;140(1-2):88–101. 10.1016/j.agee.2010.11.015

[66] Dusseux P, Gong X, Hubert-Moy L, Corpetti T. Identification of grassland management practices from leaf area index time series. Journal of Applied Remote Sensing. 2014;(8).

[67] Blanke JH, Lindeskog M, Lindström J, Lehsten V. Effect of climate data on simulated carbon and nitrogen balances for Europe. Journal of Geophysical Research: Biogeosciences. 2016;121(5):1352–1371.

